# Preoperative fasting and acute pain after cesarean section: evaluating clinical translation and generalizability of ERAS-aligned evidence

**DOI:** 10.1097/MS9.0000000000005149

**Published:** 2026-05-13

**Authors:** Pulkit Kapoor, Shaun Nevil, Shahd Khatib, Anton Charles

**Affiliations:** a Internal Medicine, Punjab Institute of Medical Sciences, Punjab, India; b Internal Medicine, ESIC Medical College & PGIMSR, Bengaluru, India; c Faculty of Medicine, Al-Quds University, Jerusalem, Palestine

*Dear Editor,*

Postoperative pain following cesarean section remains a clinically significant challenge, affecting maternal recovery, breastfeeding initiation, and the risk of chronic pain development, with poorly controlled acute pain reported in up to 80% of postoperative patients globally^[^1^]^. Preoperative fasting, a cornerstone of perioperative care, is endorsed by Enhanced Recovery After Surgery (ERAS) guidelines as a safe and beneficial practice, recommending abstinence from solids for up to 8 hours and clear fluids for 2 hours prior to surgery^[^2^]^. Its primary intent is aspiration risk mitigation; however, its potential analgesic implications have remained largely unexplored. We read with great interest the retrospective cohort study by Xu *et al*^[^3^]^, recently published in this journal, which addresses this precise knowledge gap by examining whether ERAS-compliant preoperative fasting adherence is associated with reduced 24-hour Visual Analog Scale (VAS) pain scores following cesarean section.

The study enrolled 329 women at a single Chinese tertiary center between March and December 2024. Women adhering to ERAS fasting guidelines demonstrated significantly lower mean 24-hour VAS scores compared to non-adherent women in both unadjusted (*β* = −0.6; 95% CI: −1.2, −0.1; *P* = 0.029) and partially adjusted analyses. The mechanistic premise that appropriate fasting may attenuate inflammatory mediator release, reduce central sensitization, and restore gastrointestinal homeostasis is biologically plausible, given established links between preoperative nutritional state and systemic inflammatory response^[^4^]^. Subgroup analyses further suggested consistent directional effects across categories, including ASA II status, age, gestational diabetes, hypertension, and intraoperative blood loss, reinforcing the internal coherence of this association.

Nonetheless, several considerations bearing on clinical translation and generalizability merit attention. First, the observed 0.6-point VAS reduction, while statistically significant, falls below the widely accepted minimal clinically important difference (MCID) of 1.0 to 2.0 points for acute pain on the VAS^[^5^]^. Without accompanying data on opioid rescue requirements, time to ambulation, breastfeeding outcomes, or patient-reported recovery experience, the functional significance of this difference remains uncertain. Clinical translation demands that statistical significance be accompanied by demonstrable improvement in patient-centered outcomes, and this gap represents an important limitation in interpreting the practical value of the finding.

Second, and perhaps most critically, the association attenuated to non-significance following full multivariable adjustment inclusive of epidural morphine use (*β* = −0.5; *P* = 0.137). The fasting-adherent group received 2 mg epidural morphine at substantially higher rates (65.6% versus 45.5%; *P* < 0.001), introducing a strong potential confounder or mediator that may account for a meaningful portion of the observed analgesic benefit. Epidural morphine is among the most effective analgesic strategies available following cesarean delivery, with a well-documented capacity to reduce postoperative pain scores and opioid requirements^[^6^]^. Disentangling the independent contribution of fasting from differential opioid exposure will require prospective designs that standardize analgesic protocols across adherence groups.

Third, the mechanistic hypothesis linking fasting to reduced postoperative pain mediated through inflammatory modulation is not directly supported by biomarker data in this study. No serial measurements of interleukin-6, tumor necrosis factor-alpha, or other inflammatory mediators were performed, nor were neuroendocrine stress markers or metabolic indices assessed. Although experimental evidence supports a modulatory effect of short-term fasting on systemic inflammation^[^4^]^, extrapolating this to a clinically meaningful analgesic mechanism requires empirical validation in prospective obstetric cohorts. Furthermore, patient-reported fasting duration is subject to recall and social-desirability bias, and objective verification, such as antral content assessment via gastric ultrasound, was not employed, introducing the possibility of non-differential misclassification^[^7^]^.

Apart from these limitations, we commend the team for illuminating a clinically motivated and underexplored association within perioperative obstetric care. Preoperative fasting is a modifiable, inherently safe, and low-cost parameter, and even modest analgesic contributions, if prospectively confirmed, could hold public health relevance given that cesarean deliveries now account for over 21% of all births worldwide^[^8^]^. We advocate for future multi-center prospective trials that standardize analgesic protocols across fasting adherence groups, objectively verify fasting duration, incorporate longitudinal VAS assessments and patient-reported outcome measures (PROMs), and enroll diverse populations, including emergency cases and higher-acuity patients. Until such confirmatory evidence is available, the integration of preoperative fasting as an analgesic adjunct within obstetric ERAS pathways should be regarded as hypothesis-generating, and its adoption into clinical practice pathways should proceed with appropriate caution and evidence-based oversight.

## Data Availability

Not applicable.
